# Exploring the Binding Specificity of Siglec-Derived Peptides to Human Primary Amine Oxidase Using an Integrative Approach to Ligand Epitope Mapping

**DOI:** 10.34133/csbj.0195

**Published:** 2026-08-05

**Authors:** Marion Alix, Mikael Londen, Gabriela Guédez, Ana Sofia Grosso, Filipa Marcelo, Eurico Cabrita, Tiina Annamaria Salminen

**Affiliations:** ^1^ Structural Bioinformatics Laboratory, Biochemistry, Faculty of Science and Engineering, Åbo Akademi University, Tykistökatu 6A, 20520 Turku, Finland.; ^2^ InFLAMES Research Flagship Center, Åbo Akademi University, 20520 Turku, Finland.; ^3^ UCIBIO—Applied Molecular Biosciences Unit, Department of Chemistry, NOVA School of Science and Technology, 2829-516 Caparica, Portugal.; ^4^ Associate Laboratory i4HB—Institute for Health and Bioeconomy, NOVA School of Science and Technology, 2829-516 Caparica, Portugal.

## Abstract

Human primary amine oxidase (AOC3) is a membrane-anchored protein expressed on the luminal surface of the vascular endothelium at sites of inflammation. It binds via protein–protein interactions to the CE-loops of the C2_2_ domain in Siglec-9 and Siglec-10, but the exact mechanism of interaction is unknown on an atomic level. The AOC3 binding to a radiolabeled Siglec-9 peptide can be used to image inflammation by positron emission tomography (PET). In the present study, a combination of computational and wet lab experiments is used to map the binding of the original and designed shorter Siglec-derived peptides to AOC3, to determine what features in the CE-loop are important for the interaction. To select the peptides for wet lab studies, docking and molecular dynamics simulations were first conducted to predict the binding mode of the peptides in the AOC3 active site channel, and MMGBSA calculations to predict binding energies. Selected peptides were further analyzed through microscale thermophoresis to affirm binding and saturation transfer difference NMR (STD-NMR) to determine the binding epitope to AOC3. Our results unveil that the shorter cyclic Siglec-9 and Siglec-10 peptides derived from the CE-loop bind to AOC3. Our results summarize that a tryptophan and an arginine in both peptides are important for the AOC3 interactions, but their binding mode differs slightly. Our results suggest that the designed shorter Siglec-9 peptide could be advantageous in PET imaging of inflammation and the binding properties of the Siglec-10 peptides hint a biological role for the CE-loop of the C2 domains in Siglec-10.

## Introduction

Human health is dependent on a functioning inflammatory response as part of our defense against pathological aggressions, such as viral or bacterial infections. However, overactivity and oversensitivity of the inflammatory response can lead to chronic autoimmune conditions such as rheumatoid arthritis and chronic obstructive pulmonary disease. Furthermore, the inflammatory response is often utilized by different cancer types to promote tumor survival and proliferation, and tumor-promoting inflammation is considered to be one of the hallmarks of cancer [1]. During inflammatory conditions, the expression of human primary amine oxidase (AOC3, UniprotKB Q16853; also called vascular adhesion protein-1) is up-regulated on the luminal surface of the vascular endothelium [2]. Some leukocytes, for example, CD4^+^ and CD8^+^ T lymphocytes, B lymphocytes, and granulocytes, move through the bloodstream via a rolling motion along the vascular endothelium [3]. This motion is in part dependent on interactions between AOC3 and the sialic-acid immunoglobulin-like lectin 9 (Siglec-9) receptor on leukocytes. The transient AOC3–Siglec-9 complex relies on a multivalent binding mechanism via protein–protein and protein–glycan interactions [4]. During inflammatory reactions, there are changes to the luminal endothelial cells, their adhesion proteins, and their enzymatic activity, which collectively up-regulate leukocyte trafficking [5]. AOC3 is up-regulated in some chronic inflammatory diseases, such as liver cirrhosis and hepatocellular carcinoma [6,7], lung inflammation [8], diabetes [9], and psoriasis and rheumatoid arthritis [10]. Therefore, targeting the interactions between Siglec-9 and AOC3 is an essential step in learning how to detect chronic inflammation and prevent its consequences.

AOC3 is an extracellular protein from the copper-containing amine oxidase family [11]. It is a heart-shaped, homodimeric protein of 180 kDa and is found in both membrane-anchored and soluble isoforms [9,12]. AOC3 has 2 functions, enzymatic activity and adhesion, both affecting the regulation of inflammatory responses [3]. The enzymatic function of AOC3 catalyzes the oxidative deamination of various primary amines to form the corresponding aldehyde, ammonia, and hydrogen peroxide [11]. The hydrogen peroxide resulting from enzymatic activity also regulates the lymphocyte rolling by inducing P-selectin expression [13]. The adhesive function helps lymphocytes with their extravasation through blood vessel walls and into inflamed tissues [14]. Inhibiting either of its functions with antibodies or enzymatic inhibitors has been shown to attenuate inflammatory response in animal models [15].

Each monomer of AOC3 has an N-terminal transmembrane helix and 3 extracellular domains: D2, D3, and D4 [12]. The catalytic site in the D4 domain is characterized by a tyrosine residue that is posttranslationally modified to trihydroxyphenylalanine-quinone, commonly known as topaquinone (TPQ) [11]. The autocatalytic modification of Y471 to TPQ is mediated by a copper ion coordinated by 3 histidine residues (H520, H522, and H684 in AOC3) [12]. The copper ion also steers TPQ into its inactive and active conformations: on and off copper [16]. The specificity pocket in the D3 domain is created by D180, M211, T212, and T210 in human AOC3 [17], allowing the binding of imidazole and triazole moieties [18,19]. Rat and mouse AOC3 have mutations in the specificity pocket [20,21], causing reduced binding of human AOC3 targeted inhibitors to rodent AOC3 [18] and problems in animal models for development of in vivo AOC3-targeted imaging of inflammation [22]. Eleven structures of AOC3 are available in the Protein Data Bank (PDB), with resolutions ranging from 3.8 to 2.50 Å [23]. Inhibitors such as imidazole [19] or pyridazinones [18] are bound in 6 structures, and the crystal structures prove the presence of 12 N-glycosylation sites in the AOC3 dimer.

The Siglec family is a wide family of proteins expressed in many different species. Different Siglecs have a preference for specific sialylated glycans [24]. In human, 14 Siglecs are found and can be further subdivided into conserved (Siglec-1, -2, -4, and -15) and CD33-related (Siglec-3, -5, -6, -7, -8, -9, -10, -11, -12, and -14). Orthologs of the conserved Siglecs are present across species, while the CD33-related Siglecs have evolved rapidly and differ between species. A total of 9 Siglecs are found in mice, the 4 conserved Siglecs and 5 CD33-related Siglecs (Siglec-E, -F, -G, and -H and Siglec-3) [25,26].

Siglec-9 is a 3-domain protein: The distal N-terminal V-set domain is followed by a C2_1_ and then a C2_2_ domain, a short transmembrane helix, and an intracellular immunoreceptor tyrosine-based inhibitory motif-like (ITIM-like) signaling motif. The V-set domain of Siglec-9 has a conserved arginine, R120, which is critical for sialic acid binding [27]. Siglec-9 is found on the surface of certain leukocytes, more specifically on monocytes, neutrophils, classical dendritic cells, natural killer cells, and certain subsets of T cells [25]. During the extravasation cascade, leukocytes expressing Siglec-9 on their surface roll along the vascular endothelium. At the sites of inflammation where AOC3 is expressed on the endothelium, Siglec-9 binds via its V-domain to the AOC3-attached sialoglycans and a specific CE-loop of the C2_2_ domain (Siglec-9-C2_2_) mediates the protein–protein interaction with AOC3. Both steps are important for efficient AOC3 function during the leukocyte extravasation [4].

While the interaction between AOC3 and Siglec-9 has been studied extensively, Siglec-10 was actually the first binding partner of the Siglec family that was identified. Kivi et al. [28] used a phage display assay to identify a peptide with high similarity to the CE-loop of the Siglec-10 C2_2_ domain. Further experiments confirmed the interaction between Siglec-10 and AOC3 and suggested that the arginine residue in the CE-loop of Siglec-10-C2_2_ might act as a substrate for AOC3 enzymatic activity. However, the interaction between Siglec-10 and AOC3 has not been studied further since. Interestingly, Siglec-9-C2_2_ shares higher sequence identity with the C2_3_ domain of Siglec-10 than with Siglec-10-C2_2_. Moreover, the 2 arginines in the CE-loop of Siglec-9-C2_2_ reported to be important for the interaction with AOC3 are not conserved between Siglec-9-C2_2_ (R284 and R290) and Siglec-10-C2_2_ (R293) [4,28]. As a summary, both Siglec-9 and Siglec-10 are known to interact with AOC3, but the atomic basis of the interactions remains unknown, and the known facts hint at the possibility of different binding modes for Siglec-9-C2_2_ and Siglec-10-C2_2_.

The protein–protein interaction between AOC3 and Siglec-9 can be utilized to visualize inflammation [21]. The radiolabeled 17-amino acid peptide, derived from residues 283 to 297 in the CE-loop of Siglec-9-C2_2_ (CARLSLSWRGLTLCPSK) and cyclized via a disulfide bridge, has provided targeted in vivo imaging of AOC3 at the sites of inflammation using positron emission tomography (PET) in several preclinical disease models (e.g., autoimmune myocarditis [22] and inflammatory bowel disease [29]). The peptide, hereafter called Sig9-PET, has shown its potential as a PET tracer in the clinical trials of imaging rheumatoid arthritis in human [30].

However, the Sig9-PET peptide is longer than the original phage peptide through which the interaction first was identified. Moreover, it is uncommonly long compared to other peptides used in clinics and, thus, a shorter and smaller Siglec-9-derived peptide could have better absorbability, accessibility, selectivity, specificity, and safety as has been reported for other peptides [31]. To study how the amino acid composition and the length of the peptide affect its interactions with AOC3, we started with a systematic in silico analysis of peptides derived from the CE-loop of C2_2_ in Siglec-9 and C2_2_ and C2_3_ in Siglec-10. In addition to the previously studied long Sig9-PET, its arginine variants (Sig9-R1A, Sig9-R2A) [21,32], and the short phage peptides [21], we designed and characterized shorter cyclic Siglec-9-C2_2_, Siglec-10-C2_2_, and Siglec-10-C2_3_ peptides corresponding to the original phage peptides. In this study, we first predicted the binding properties of the Siglec peptides to AOC3 using computational methods including peptide docking, molecular dynamics simulations (MDs), and molecular mechanics with general born surface area (MMGBSA). Thereafter, the binding properties of the selected peptides were further analyzed with the experimental methods saturation transfer difference nuclear magnetic resonance (STD-NMR) and microscale thermophoresis (MST). This study shed light on the role of the individual residues in the short peptides derived from the CE-loops of Siglec-9 and Siglec-10 and, importantly, unveiled the unique tryptophan residue of the Siglec-9-C2_2_-derived peptide as a new key determinant of AOC3 binding.

## Materials and Methods

### Experimental and technical design

**Fig. 1.**
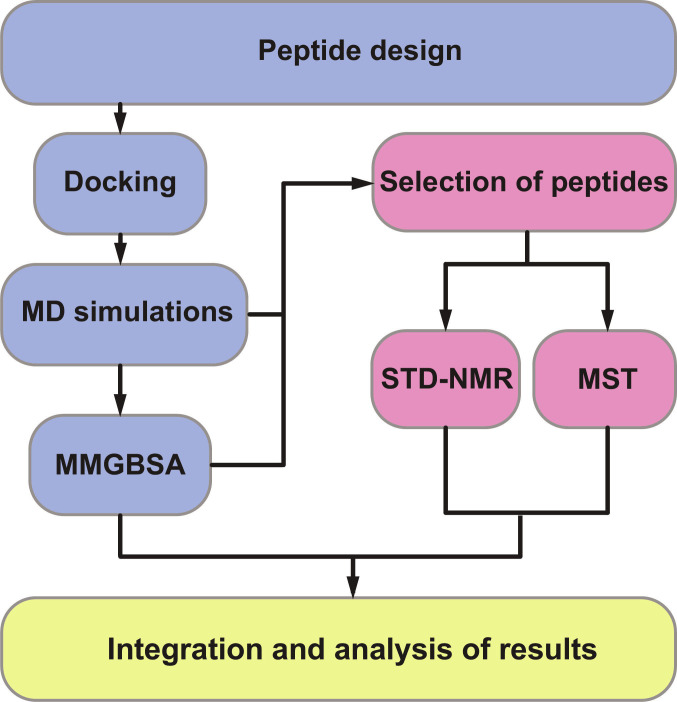
Flowchart illustrating the entire experimental design to understand peptide binding to AOC3.

Figure 1 illustrates the complete experimental design of this article, which started with peptide design and in silico methods to characterize the Siglec-9 and Siglec-10 peptide interactions with the AOC3 protein. The in silico study, performed with docking, MDs, and MMGBSA calculations, led to the selection of peptides to further characterize their interactions by the in vitro methods STD-NMR and MST. Finally, the results are analyzed together.

### Siglec peptide design

For this study, we first analyzed the AOC3 binding of several cyclic Siglec peptides, computationally to select peptides for experimental analysis. The original 10-amino acid peptides from the phage display assay, Sig9-phage [32] and Sig10-phage [28], were included in the study as positive controls (Table 1). The 14-amino acid cyclic part (without DOTA-tag and linker) of the PET-tracer peptide (Sig9-PET) derived from the CE-loop of Siglec-9-C2_2_ [32] was used as a starting point. We also studied the mutants of Sig9-PET, named Sig9-R1A and Sig9-R2A, which were previously shown to have lower affinity binding to AOC3 (Table 1) [32].

**Table 1.** Name, sequence, and residue numbering of all peptides described, grouped by sequence similarity and size. The conserved residues that correspond to the WRG motif in Sig9 and the conserved W and R residues in Sig10 and SigE are in bold font.

Residue nr	−4	−3	−2	−1	1	2	3	4	5	6	7	8	9	10
Sig9-PET	C	A	R	L	S	L	S	**W**	**R**	**G**	L	T	L	C
Sig9-R1A	C	A	A	L	S	L	S	**W**	**R**	**G**	L	T	L	C
Sig9-R2A	C	A	R	L	S	L	S	**W**	A	**G**	L	T	L	C
Sig9-phage					C	V	K	**W**	**R**	**G**	V	V	V	C
Sig9-C2_2_					C	L	S	**W**	**R**	**G**	L	T	L	C
Sig10-C2_3_					C	**W**	T	Q	**R**	**G**	Q	V	L	C
Sig10-phage					C	**W**	S	F	**R**	N	**R**	V	V	C
Sig10-C2_2_					C	**W**	V	L	Q	N	**R**	V	L	C
SigE					C	**W**	S	**W**	D	N	L	T	L	C

Closer analysis of these peptides reveals that there are only 3 residues that are conserved between Sig9-phage and Sig9-PET, the ^4^WRG^6^ sequence, which corresponds to the C-terminal part of Sig9-PET peptide (Table 1). The ^4^WRG^6^ motif is not conserved in any other Siglecs than the C2_2_ domain of Siglec-9 [19]. Since shorter peptides might have benefits in biomedical imaging, we designed 10-amino acid peptides corresponding to the phage peptides: Sig9-C2_2_, covering the C-terminal part of the 14-amino acid Sig9-PET, and Sig10-C2_2_, corresponding to Sig10-phage. We also studied Sig10-C2_3_, covering the corresponding residues in the C2_3_ domain of Siglec-10 and having a higher sequence identity to Sig9-C2_2_ than Sig10-C2_2_. Additionally, a corresponding peptide was also derived from the C2_2_ domain of mouse Siglec-E (SigE), which is the closest homolog to Siglec-9 found in mice. The SigE peptide serves as a negative control in our study since it does not have any arginines and, thus, cannot interact with mouse AOC3 [32]. Sig9-phage, Sig9-C2_2_, SigE, Sig10-C2_2_, and Sig10-C2_3_ were purchased from PSL Peptide Specialty Laboratories GmbH (Germany) and used for experimental studies. The AlphaFold3 models for Siglec-9-C2_2_ and Siglec-10-C2_2_ were created to locate the 14- and 10-amino acid peptides in the CE-loop of the C2 domains [33] and visualized using PyMOL (The PyMOL Molecular Graphics System, version 2.5.7, Schrödinger, LLC).

### In silico interaction analysis

All peptides described in Table 1 were explored in the initial computational experiments. AOC3 structure (PDB-ID:4BTY) [18] was prepared for docking with standard parameters used for calculation of vibrational modes (5 modes, 20 frames, and maximal vibrational of 10 Å) implemented in Maestro Schrödinger release 2019-4 (Maestro, Schrödinger, LLC, New York, NY, 2019). A fragment of the pyridazinone ligand (Fig. S1) in chain B of the structure with the wider active site cavity was used to define the center of docking using CSD Gold within Hermes (GOLD © 2022 CCDC software Ltd Jones et al 1997 Hermes © 2022 CCDC software). Peptides were modeled and energy minimized using the 3-dimensional (3D) builder tool feature within Maestro Schrödinger release 2019-4.

Docking poses were visually analyzed using PyMOL (The PyMOL Molecular Graphics System, version 2.5.7, Schrödinger, LLC), and the representative poses of each cluster were subjected to MDs. MDs were run using the Amber 20 package with the ff14SB force field, and each complex was immersed in a water box of 10 Å using the TIP3P water model and neutralized with counter ions (Na^+^, Cl^−^) [34–36]. A 2-step minimization was performed, the first only hydrogen minimization and the second all-atom minimization, respectively, using 1,000 and 10,000 steps of steepest descent and continuing with conjugate gradient. Heating was performed with the Langevin-dynamic thermostat from 0 to 300 K with periodic boundary conditions and constant volume. Independent equilibrations were done with constant pressure, isotropic position scaling at 1 atm pressure for 20 ns for each duplicate. Production trajectory was run with 2 initial velocities, in duplicates for 100 ns, with a time step of 0.002 ps. Finally, MMGBSA was calculated using AmberTools20 [34] using 1 frame every 100 frames from the production file (100 frames in total), with the atomic radii of 2. MMGBSA results are reported in kcal/mol, and the studied peptides can be compared since they are of similar size. Root mean square deviation (RMSD) and distance analysis were done using CPPTRAJ distance calculation [37] and express that the system is at equilibrium for the given simulated timescale. The results are reported in Å.

### Binding assays

To validate the findings of the computational experiments, 4 of the shorter peptides were chosen for binding assays: the original Sig9-phage peptide (positive control), the corresponding human Sig9-C2_2_ designed in this work, Sig10-C2_2_, which is similar to the Sig10-phage peptide, and mouse SigE (negative control). AOC3 protein, purchased from PeproTech (amino acid residues 27 to 763), was labeled with RED-NHS dye (NT-647-NHS) using the Monolith NT Protein Labeling Kit (product number MO-L001, NanoTemper Technologies, Germany) according to provided instructions, and the binding of the Siglec peptides to AOC3 was detected using MST in a solution of phosphate-buffered saline (PBS) pH 7.4 [38]. The Siglec-9 peptides (phage, human, and mouse peptides) were mixed with labeled AOC3 (50 to 60 nM final concentration) in zero-background glass capillaries (product number MO-AK002; NanoTemper Technologies, Germany) as 12-point serial dilution. The final concentration of the peptides ranged from 130 μM for Sig9-C2_2_ and 160 μM for Sig9-phage and mouse SigE. For Sig10-C2_2_, a 14-point serial dilution starting at 231.25 μM and a target concentration of 50 nM AOC3 was used. Samples were measured with a Monolith.NT115 instrument (NanoTemper Technologies) using medium MST power, 35% to 50% excitation power, and Nano-RED excitation type at 25 °C. The resulting data were analyzed using MO. Affinity Analysis software (NanoTemper Technologies) and GraphPad Prism (version 8.0; GraphPad Software, USA). The dissociation constants were calculated from 3 or 4 independent measurements.

### Determination of peptide binding epitopes by STD-NMR

The interactions between Sig10-C2_2_ (corresponding to Sig10-phage), Sig10-C2_3_ (most similar to Sig9-C2_2_), and Sig9-phage (positive control) and AOC3 were further studied by STD-NMR [39] to determine the binding epitope of the interaction. The peptide choice was limited by their solubility in PBS pH 7.4. The Siglec-10 peptides were chosen because they are rarely studied and could represent different binding modes when compared to the better understood interaction with the Siglec-9-derived peptides.

For the assignment of the peptides, a combination of 2D total correlation spectroscopy (TOCSY), nucelar Overhauser effect spectroscopy (NOESY), rotating-frame nucelar Overhauser effect spectroscopy (ROESY), and ^13^C heteronuclear single-quantum correlation spectroscopy (^13^C-HSQC) experiments was used. The assignment of the Sig9-phage and Sig10-C2_2_ peptides was performed using a 500-MHz Bruker Ascend spectrometer equipped with a Prodigy cryoprobe CRPN2-TR-^1^H&^19^F/^13^C/^15^N-5mm-EZ and a 600-MHz Bruker Avance III spectrometer equipped with a 5-mm inverse detection triple-resonance z-gradient cryogenic probe, head (CP TCI), while the assignments for Sig10-C2_3_ were done with a 500-MHz Bruker Avance-III NMR spectrometer with a Prodigy BBO cryoprobe. The peptides were dissolved to concentrations of approximately 1.5 to 2.5 mM in water, with a H_2_O:D_2_O ratio of 9:1. All measurements were carried out at 298 K. The resonance of 2,2,3,3-tetradeutero-3-trimethylsilylpropionic acid (TSP) was used as a chemical shift reference in the ^1^H-NMR experiments [δ TSP = 0 part per million (ppm)]. All spectra were processed and calibrated with TopSpin 4.3.0, and the assignments were made using the software CARA (Computer Aided Resonance Assignment) version 1.9.1.7. Chemical shifts of the peptide protons are shown in Table S2.

The peptides were characterized again in the STD-NMR conditions. The STD-NMR experiments for Sig9-phage and Sig10-C2_2_ were performed in a 600-MHz Bruker Avance III spectrometer equipped with a 5-mm inverse detection triple-resonance z-gradient cryogenic probe, head (CP TCI), whereas the Sig10-C2_3_ peptide, as well as a bovine serum albumin (BSA) control experiments, was done with a 500-MHz Bruker Avance III NMR spectrometer with a Prodigy BBO cryoprobe. The samples were prepared using a 1:100 ratio defined by the ratio of peptide to possible binding sites (2 per AOC3 dimer), in this case 2.5 μM of AOC3 and 500 μM of peptide, except for the STD-NMR with Sig10-C2_3_ (1:89 molar ratio), where 445 μM of peptide was used, in a PBS D_2_O buffer pD 7.4. TSP was added as a reference for ^1^H chemical shifts. The STD-NMR experiments were acquired at 298 K using the pulse program stddiffesgp from the Bruker pulse program library with 1,728 scans and 64 K data points in a spectral window of 9,014.42 Hz centered at 2,821 Hz. Selective saturation (on-resonance) was performed by irradiating at −0.5 ppm using a series of 40 Eburp2.1000-shaped (from Bruker-shaped pulses library) 90° pulses (50 ms) for a total saturation time of 2 s and a relaxation delay of 4 s. For the reference spectrum (off-resonance), the samples were irradiated at 100 ppm. Proper control experiments were performed for each ligand in the absence of protein, and the residual STD signals observed were subtracted from the STD experiment containing protein. The STD spectrum (*I*_STD_) was obtained by subtracting the on-resonance spectrum (*I*_on_) to the off-resonance spectrum (*I*_off_). The % of STD (*I*_STD_/*I*_off_ × 100) was estimated by comparing the intensity of the signals in the STD spectrum (*I*_STD_) with the signal intensities of the reference spectrum (*I*_off_). To determine the STD-derived epitope map, the relative % of STD was calculated by setting to 100% the STD signal of the proton with the highest STD intensity and calculating the others accordingly.

## Results

### In silico interaction analysis of CE-loop peptides derived from Siglec-9 and Siglec-10

**Fig. 2.**
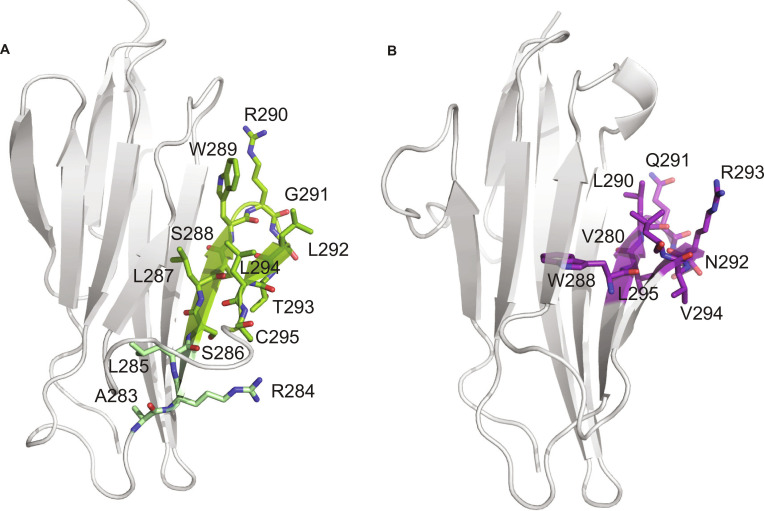
The CE-loops of (A) the Siglec-9-C2_2_ and (B) Siglec-10-C2_2_ domains. The cyclic Sig9-PET peptide was created by adding an N-terminal cysteine to residues A283–C295 in the CE-loop (green sticks). Visual inspection of the Siglec-9 AlphaFold3 [33] model indicates that a shorter 10-residue peptide, corresponding to residues S286–C295 (darker green), forms a circular form and allows a disulfide formation after implementation of the S286C mutation to Sig9-C2_2_. The 8 residues in the cyclic Sig10-C2_2_ peptide corresponding to the CE-loop of Siglec-10-C2_2_ are shown as deep purple sticks.

We started the in silico interaction analysis with the previously studied longer Sig9-PET [21,32] peptide originating from the CE-loop of the C2_2_ domain in Siglec-9 (Fig. 2) and its variants (Sig9-R1A and Sig9-R2A), but also studied the original phage peptides [28,32], which identified Siglec-9 and Siglec-10 as AOC3 ligands. Based on visual inspection of the CE-loop of Siglecs in the AlphaFold models, we designed novel 10-amino acid cyclic peptides originating from the C-terminal half of the CE-loop of the C2_2_ domain in Siglec-9 (Fig. 2), and the C2_2_ and C2_3_ domains in Siglec-10 that have not been studied earlier (Table 1).

**Fig. 3.**
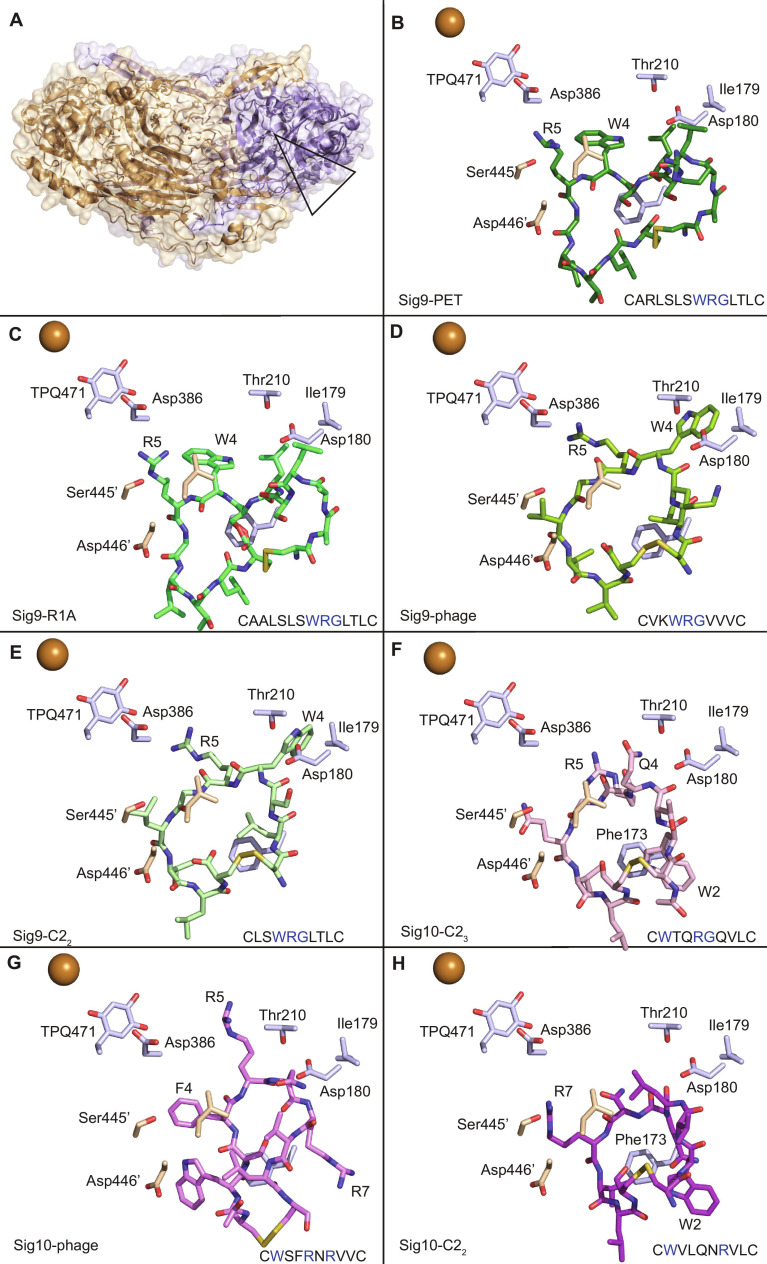
The representative binding modes of the docked peptides into AOC3. (A) Side view in cartoon and surface representation of AOC3 (chain A light cyan and chain B light blue). The binding site of peptides in chain B is illustrated by a triangle. In (B) to (F), the representative pose is shown for each peptide docking into AOC3 and the copper ion is depicted as sphere, and the AOC3 residues interacting with the peptide are shown as sticks with chain colors. (B) Sig9-PET, (C) Sig9-R1A, (D) Sig9-phage, (E) Sig9-C2_2_, (F) Sig10-C2_3_, (G) Sig10-phage, and (H) Sig10-C2_2_.

The docking protocol was implemented on the minimized cyclic peptides. However, the conformation of the 10- and 14-amino acid cyclic peptides is much more restricted compared to a linear peptide and resembles the CE-loop of Siglecs (Fig. 2). The docking clusters were named based on the binding site of peptide residues 4 (W in Sig9), L2, R5, or R7. In addition, each pose in the cluster had a similar main chain conformation. Depending on the peptide, the number of docking clusters varied from 2 to 4, and the MMGBSA results for all clusters gave negative values, suggesting potential binding (Table 2). The representative pose considered as the most probable peptide binding mode to AOC3 shown in Fig. 3 was selected from the cluster with the highest in silico affinity and having the disulfide bond toward the protein surface, which is prerequisite for the function of labeled Sig9-PET peptide.

**Table 2.** Docking results showing the number of poses per cluster and the average MMGBSA. Each column corresponds to one cluster, and each line to the peptide used in the docking study. The number of poses per cluster and the MMGBSA result are shown for each. * corresponds to the pose that shifted from a sparsely represented cluster to the dominant cluster. Bolded clusters are considered as the most probable binding mode; hence, they are depicted in Fig. 3.

Common features of the clusters	Res4 toward Asp^386^	Res4 in the specificity pocket	Res4 and R5 near ArmI	Res4 and Res5 outward	R5 or R7 outward	L2 in the specificity pocket
Sig9-PET	**3/−38.88**		1/−31.69	4/−23.28		2/−21.56
Sig9-R1A	**5/−40.62**		1/−24.86	2/−39.32		2/−29.09
Sig9-R2A	5/−22.1		1/−44.29			4/−17.25
Sig9-phage		**6/−35.53**				4/−30.58
Sig9-C2_2_		**8/**−22.63 −32.45*	1/−25.59			1/−32.45
Sig10-C2_3_		**6 /−28.18**			2/−19.61; 2/−14.68	
Sig10-phage	1/−34.0	1/−19.3	1/−11.8 **3**/**−34.32**		1/−20.75; 1/−42.65	
Sig10-C2_2_	1/−23.41	**6/−24.65**			2/−22.83	1/−22.55
SigE	1/−1.22		2/−4.12		3/−7.17; 3/−13.99; 1/−13.89	

The peptide dockings generated 10 binding poses to chain B of the AOC3 dimer (Table 2). Thereafter, each cluster underwent MDs to evaluate the stability of the binding mode. Moreover, the free energies of binding (MMGBSA) were calculated to predict which peptide binding mode has the strongest binding affinity with AOC3, as MMGBSA is an approximation of free energies of binding and a more negative value indicates stronger binding.

**Fig. 4.**
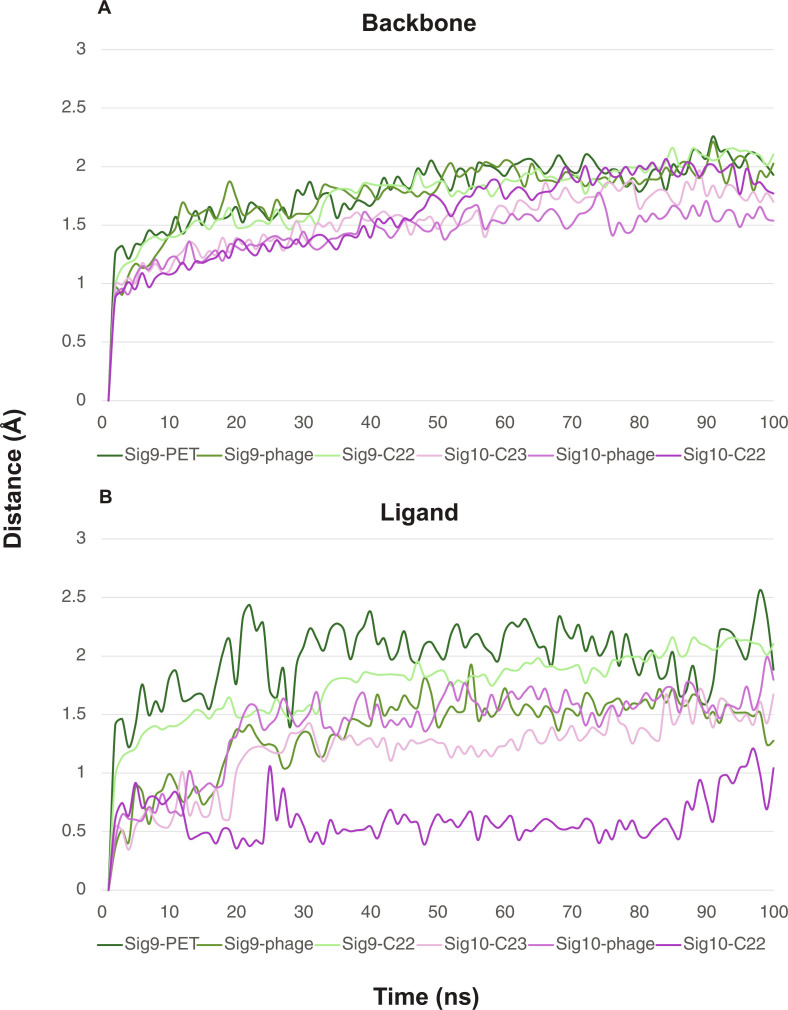
Root mean square deviation (RMSD) of protein backbone (A) and peptide backbone (B) during MDs of docking representatives. Each peptide is represented using the same color as the docking results (Fig. 3).

The major clusters, e.g., Res4 toward Asp^386^ and Res4 in the specificity pocket, were stable in the MDs (Fig. 4A), whereas the minor clusters, Res4 and R5 near ArmI, showed movements. The Sig9-C2_2_ peptide equilibrated with W4 in the specificity pocket, and the other clusters no longer existed. Sig10 poses stayed stable during MDs, with W2 forming hydrophobic interactions with L177 in Sig10-C2_2_ and Sig10-C2_3_ (Fig. S4D and F), while Sig10-phage forms transient hydrophobic interactions with F415 and I417 (Fig. 3G and Fig. S2E). Analysis of the peptide RMSD during the MDs shows distances mainly under 2.5 Å and reaching a stable conformation for all peptides before 20 ns (Fig. 4B). These distances show the stability of the docking poses of the selected representative clusters, confirming that the minimized initial peptide structure and the sampling during the docking experiments are sufficient to find a stable pose for our Siglec peptides on AOC3.

The cluster of Sig9-PET peptide docking results with 3 poses had the strongest binding affinity (−38.88 kcal/mol) and was chosen as the representative binding mode (Fig. 3B), whereas the cluster with 4 poses was predicted to have weaker binding to AOC3 (−23.28 kcal/mol). The major cluster for Sig9-R2A, lacking R5, had a lower predicted binding affinity (−22.1 kcal/mol) than the major cluster of Sig9-R1A (Fig. 3C), which still had the key R5 (−40.62 kcal/mol), emphasizing the important role of R5, which was also present in the original Sig9-phage peptide.

The docked binding mode of the Sig9-PET peptide differs slightly from that of the Sig9-phage (Fig. 3D) and Sig9-C2_2_ peptides (Fig. 3E), since its W4 interacts with N470 via hydrogen bonds (Fig. S2A) and occupies the cavity leading to the active site instead of R5. The R5 in Sig9-PET is located below ArmI originating from chain A of the AOC3 dimer and interacts with N470 and D472 via ionic interactions and hydrogen bonds (Fig. S2A). The Sig9-phage and corresponding Sig9-C2_2_ peptide both had a clear major cluster with 6 and 8 poses, respectively, with the strongest predicted binding affinity as well. Their binding mode was predicted to be similar: Tryptophan W4 interacts with the specificity pocket of AOC3 lined by residues T210, D180, and I179, and forms pi-interactions with Y176, and with D180 for Sig9-phage (Fig. S2B and C). The sole arginine R5 forms ionic interactions with the catalytic aspartate, D386 (Fig. 3B and C and Fig. S2B and C). The major cluster for the Sig10-C2_3_ (Fig. 3F) and Sig10-C2_2_ (Fig. 3H) peptides had Q4 in the specificity pocket and W2 forming hydrophobic interactions with L177 and with F173 for Sig10-C2_2_ (Fig. S2D and F). Sig10-C2_2_ and Sig10-C2_3_ had MMGBSA values between −28.18 and −24.65 kcal/mol in their most represented cluster, in accordance with a slightly different binding mode.

The peptides with 2 arginines, R2 and R5 in Sig9-PET (Fig. 3B) and R5 and R7 in Sig10-phage (Fig. 3G), displayed higher affinities across multiple clusters, implying that the presence of 2 arginine residues allows for several binding modes (Table 2). In contrast to all other peptides, the negative control peptide SigE created 5 different clusters, and only one of them corresponds to the cluster highly represented by the other peptides. The SigE clusters underwent large movements during MDs, and most poses shifted away from the binding site. Moreover, the SigE peptide had affinities ranging from −14.53 to −1.22 kcal/mol, which are much lower values than those of the other peptides, and it also had lower decomposition per residue values (Table 3).

**Table 3.** Decomposition per residue for each peptide based on MMGBSA values

MMGBSA per residue (kcal/mol)	−4	−3	−2	−1	1	2	3	4	5	6	7	8	9	10
Sig9-PET	C^1.2^	A^−0.4^	R^−1.0^	L^−2.4^	S^−0.7^	L^−4.5^	S^−0.1^	W^−6.0^	R^−16.6^	G^−2.1^	L^−5.8^	T^−0.3^	L^−1.4^	C^2.4^
Sig9-R1A	C^0.7^	A^−0.1^	A^−0.4^	L^−2.7^	S^−0.7^	L^−5.8^	S^−0.9^	W^−8.4^	R^−16.2^	G^−3.5^	L^−3.5^	T^−3.3^	L^−0.6^	C^2.5^
Sig9-R2A	C^−0.3^	A^0.0^	R^−4.6^	L^−0.9^	S^0.4^	L^−4.0^	S^0.1^	W^−6.9^	A^−3.2^	G^−1.5^	L^−1.5^	T^−0.2^	L^−0.3^	C^1.9^
Sig9-phage	/	/	/	/	C^3.4^	V^−3.6^	K^−3.1^	W^−6.7^	R^−15.7^	G^−0.9^	V^−3.4^	V^−4.0^	V^−2.0^	C^0.9^
Sig9-C2_2_	/	/	/	/	C^3.7^	L^−3.5^	S^−0.1^	W^−7.2^	R^−15.1^	G^−0.7^	L^−4.2^	T^−3.4^	L^−2.8^	C^2.5^
Sig10-C2_3_	/	/	/	/	C^2.0^	W^−0.7^	T^−0.5^	Q^−1.7^	R^−10.1^	G^0.1^	Q^−5.2^	V^−4.6^	L^−6.1^	C^3.2^
Sig10-phage	/	/	/	/	C^0.6^	W^−3.3^	S^−4.4^	F^−3.9^	R^−4.8^	N^−3.0^	R^−6.0^	V^−5.4^	V^−2.4^	C^1.5^
Sig10-C2_2_	/	/	/	/	C^1.9^	W^−2.2^	V^−3.0^	L^−7.0^	Q^−0.6^	N^−1.3^	R^−8.7^	V^−2.4^	L^−2.9^	C^1.4^
SigE	/	/	/	/	C^0.8^	W^−1.4^	S^−0.3^	W^−3.7^	D^2.4^	N^0.3^	L^−0.1^	T^−1.1^	L^−0.2^	C^2.2^

The decomposition per residue of MMGBSA values was calculated to estimate their contribution to the binding affinity (Table 3). The results highlight the important contribution of the arginine R5 for AOC3 binding to all the Siglec-9-derived peptides. The tryptophan W4 in the Siglec-9 peptides had the second largest effect on binding based on its decomposition per residue of MMGBSA. The values for Sig10-C2_3_, which has the highest sequence identity with the CE-loop of Sig9-C2_2_, are much lower than those of Sig9-C2_2_ and in agreement with the MMGBSA values for the docking clusters. The residues in the Sig10-phage peptide have a more uniform contribution to the binding affinity. Of its 2 arginines, R7 contributes more than R5 to the binding affinity. Sig10-C2_2_ has only one arginine, R7, which along with the leucine L4 shows an effect on the binding affinity during MDs.

The 3-step approach, combining docking, MD stability analysis, and MMGBSA affinity estimations, allowed for a comprehensive assessment of the peptide interactions with AOC3, and the calculated free energies of binding predicted the strongest binding peptides. The Sig9-phage, Sig9-PET, and Sig9-C2_2_ peptides show the strongest affinities when the tryptophan residue, W4, occupies the specificity pocket of AOC3.

### Binding assays

**Fig. 5.**
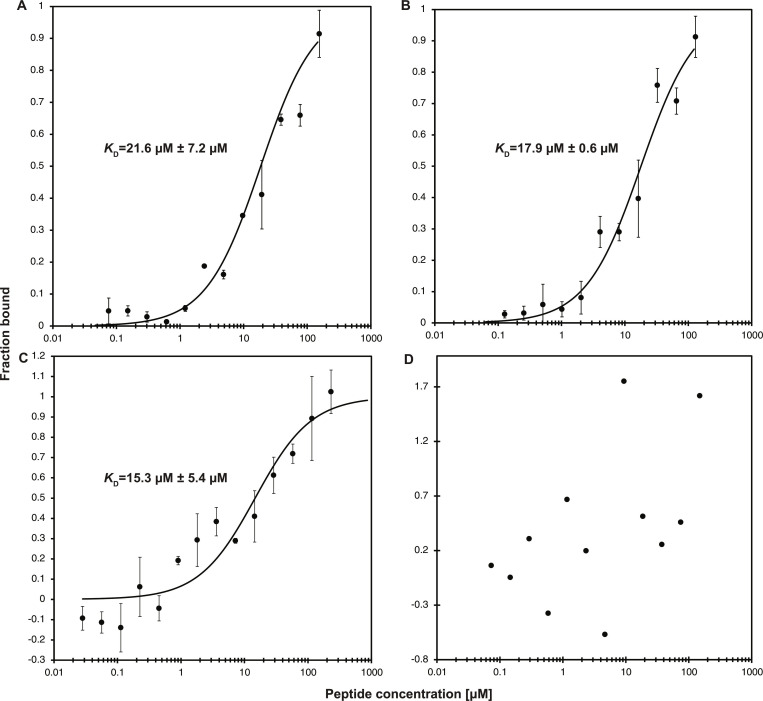
Averaged graphs of binding curves determined by replicate MST measurements of (A) Sig9-phage, (B) Sig9-C2_2_, (C) Sig10-C2_2_, and (D) SigE. The measurements result in *K*_D_ values of 21.6 ± 7.2 μM, 17.9 ± 0.6 μM, 15.3 ± 5.4 μM, and no binding, respectively. Error bars show the standard deviation of the data points.

Following computational experiments, binding assays were performed to measure the actual binding affinities (Fig. 5). The binding assays were conducted in triplicate and quadruplicate with MST and showed binding for the Sig9-C2_2_, Sig9-phage, and Sig10-C2_2_ peptides with *K*_D_ values of 17.85 ± 0.6 μM, 21.6 ± 7.2 μM, and 15.3 ± 5.4 μM, respectively. In contrast, the SigE peptide did not show any binding. The results of the individual measurements are found in Table S1.

### Determining the binding epitope of selected peptides by STD-NMR

After reviewing the results of the computational studies and confirming the binding of the selected peptides, we moved on to peptide characterization and epitope mapping using NMR techniques. Sig9-phage, Sig10-C2_3_, and Sig10-C2_2_ were chosen for this purpose. Sig9-phage was chosen due to the presence of the WRG-motif, whereas the Siglec-10 peptides were chosen due to the lack of previously available experimental data describing their interactions. In addition, they had sufficient solubility in PBS pH 7.4 to carry out NMR experiments. All 3 peptides were successfully characterized, except for residues C1 and C10, which could not always be unambiguously discerned from each other. However, these cysteines were only added to cyclize the peptides and are not derived from the Siglec sequences, so their interactions are not as interesting as the other residues of the peptides. ^1^H chemical shifts of the peptides are found in Table S2.

**Fig. 6.**
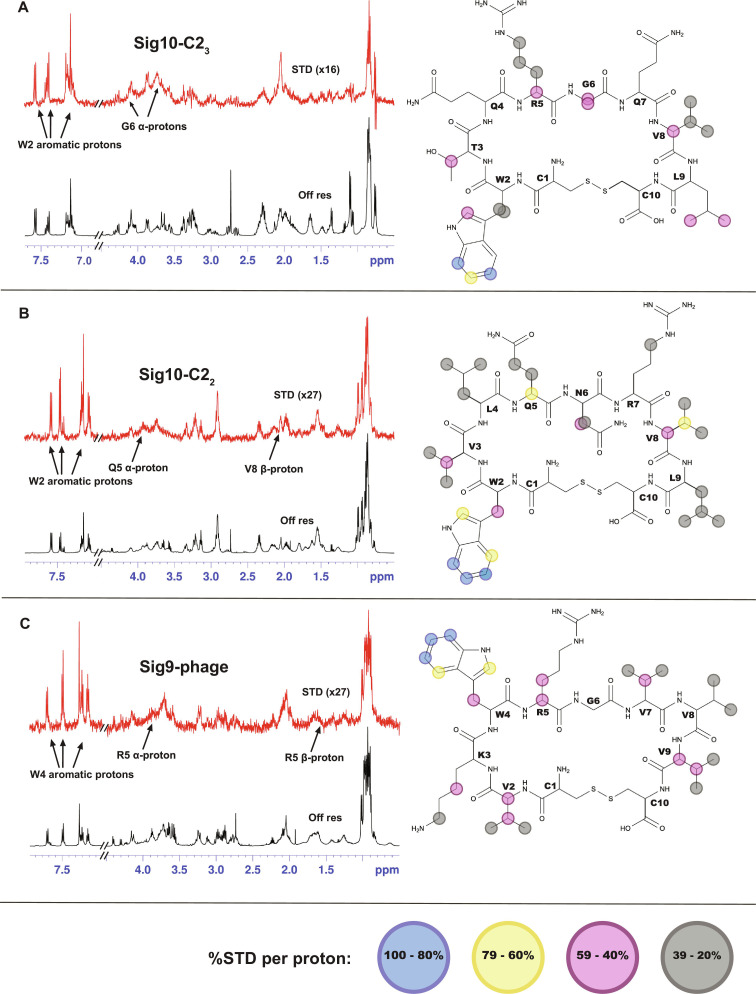
STD-NMR epitope maps of the peptides (A) Sig10-C2_3_, (B) Sig10-C2_2_, and (C) Sig9-phage. The red spectra are the STD spectra magnified to match the black off-resonance spectra. Circles indicate the STD intensities of specific protons relative to the proton experiencing the highest STD effect within each peptide. The highest STD value is normalized to 100%, and the others scaled to that value. The STD effect indicated by blue circles is 80% to 100%, yellow circles 60% to 79%, pink circles 40% to 59%, and gray circles 20% to 39%. Protons with an STD effect lower than 20% and protons that did not show a discernable signal in the NMR spectra are left without any markings.

STD-NMR was used to determine the binding epitope of the peptides. In all 3 peptides, the tryptophan residue showed the strongest interaction with the protein, despite being located on different relative locations within the peptides (Fig. 6). STD-NMR between Sig10-C2_3_ and BSA showed no STD effect on the tryptophan residue, indicating that the interaction is specific to AOC3 and not an effect of nonspecific binding of tryptophan to hydrophobic patches on proteins (Fig. S3). Other residues in the binding peptides show weaker levels of interaction spread throughout the peptide. Notably, the hydrocarbon chains of the arginine side chains in the peptides do not show any remarkable levels of interaction with the protein.

## Discussion

The interaction between Siglec-9 and AOC3 is important for the proper function of the inflammatory response, since it allows Siglec-9-expressing white blood cells to extravasate into inflamed tissues from the bloodstream [32]. Furthermore, the interaction provides the basis for a PET-tracer that is currently being investigated in clinical trials as a tool for imaging inflammation in vivo [40]. The Siglec-10 interaction with AOC3 was the first to be discovered, and it has been shown that Siglec-10 is a substrate for AOC3 [28]. This interaction has been hypothesized to be important for lymphocyte adhesion to the endothelium; however, it has not been further explored since. In this study, we have carried out extensive computational calculations and experimental validations to elucidate the importance of different amino acid residues in the binding loops of Siglec-9 and Siglec-10 in their interaction with AOC3. Shedding light on the interaction on an amino acid and atomic levels provides valuable insight into the specificity of Siglec-9 as a ligand of AOC3 and gives us a better understanding of the strengths and weaknesses of the currently used PET-tracer peptide. The shorter peptides were designed to solve the issue of the uncommonly long PET peptide by deleting weakly interacting hydrophobic residues and keeping the conserved WRG motif of Sig9-phage. We also reveal new insights about Siglec-10, whose interaction with AOC3 is known, however poorly understood.

The work started with computational studies of the 14-residue-long Sig9-PET-imaging peptide and continued back to the shorter 10-residue Siglec peptides corresponding to the phage peptides initially discovered with phage display and matching the C-terminal part of the PET peptide [28,32]. The work continued with experimental work testing shorter peptides to evaluate their binding affinity and confirm their interaction hot spots. Previously, in silico methods including docking and MDs have been used at the start of workflows to improve the development of vaccines and epitopes and the understanding of interactions [41–43]. The calculated MMGBSA values of the peptides indicate that the Siglec-9-derived peptides have stronger interactions with AOC3 than the Siglec-10 peptides across the board. The role of arginine in position 5 is highlighted by the MMGBSA decompositions with high predicted affinity shown in all cases, and the importance of the corresponding arginine residues has also been shown with mutation studies of the Siglec-9 protein previously [4]. However, the arginine residue should not be expected to show close interaction during STD-NMR experiments, as the protons are in exchange with the solvent, rendering the amine head of arginine residues invisible. Sig10-C2_3_ has also an arginine in the same position, but lacks a tryptophan in position 4, which seems to be crucial for increased binding affinity of the Siglec-9 peptides. Tryptophan residues in position 4 have high predicted affinity in all Siglec-9 peptides but not in SigE, which is the arginine-lacking negative control. The STD-NMR results of Sig9-phage are in agreement with this observation, with the tryptophan residue showing the closest interaction to the protein. In addition, in a previous study, the tryptophan in Sig9-PET has been shown to be crucial for the interaction to rat AOC3, allowing the double R/A mutated Sig9-PET control peptide to interact with the specificity pocket despite the absence of both arginine residues [22].

Our results with the longer Sig9-PET peptide used in clinical trials for PET imaging also highlight the importance of arginine in position 5, as it had a stronger predicted binding affinity than the arginine residue in position −2. The substitution of the respective arginines to alanines in Sig9-PET supports this, as the Sig9-R1A mutant exhibits higher affinity than the Sig9-R2A mutant. Arginine in position 7, as seen in Sig10-C2_2_ and Sig10-phage, also contributes relatively strongly to the binding of these peptides. Similarly to Sig10-C2_3_, these peptides have the tryptophan residue in position 2 instead of position 4. Docking and MMGBSA results show that W2 is positioned about 4 Å away from F173 and L177, which are also known to interact with the reversibly binding pyridazinone inhibitors [18]. Still, W2 in the Siglec-10 peptides do not interact with AOC3 as strongly as W4 in the Siglec-9 peptides. Similarly to the Sig9-phage STD-NMR results, Sig10-C2_3_ and Sig10-C2_2_ show tryptophan as the residue with the closest interaction. In our study, W2 in the Sig10-C2_3_ and Sig10-C2_2_ peptides were found to be important based on the MMGBSA and STD-NMR results. However, the long Siglec-9 peptides (Sig9-PET, Sig9-R1A, and Sig9-R2A) have a higher predicted affinity than Sig9-C2_2_ and Sig9-phage. Contrary to the short Siglec-9 peptides (Sig9-C2_2_ and Sig9-phage), the docking experiments with the Siglec-10-derived peptides resulted in a higher number of clusters and lower predicted binding affinity.

Combining the results from MMGBSA and STD-NMR shows that for Sig9-phage (which has a binding mode identical to Sig9-C2_2_), the results clearly support each other. The strong STD signal of residue W4 is mirrored by the high affinity predicted by MMGBSA. For Sig10-C2_2_, the result is not quite as supporting; W2, which has a strong STD signal, has an MMGBSA of only −2.2 kcal/mol, compared to L4 with −7.0 kcal/mol, but a weak STD signal. This can be attributed to the simple fact that STD-NMR and MMGBSA characterize interactions in different ways. The difference in the results leads us to believe that the binding mode identified for Sig9-phage and Sig9-C2_2_ is more definitive than the main pose identified for Sig10-C2_2_. The fact that the initial docking found more different binding modes for Sig10-C2_2_ (Table 2) supports this possibility. If there are indeed several binding modes for Sig10-C2_2_, then STD-NMR would show an average of those interactions.

Interestingly, Mitsuki et al. [44] have experimentally shown that the CE-loop of the C2_2_ domain of Siglec-7 is essential for the activation of nonapoptotic cell death of U937 monocyte cells upon clustering of the Siglec-7 molecules expressed on the cell surface. This effect could not be replicated when the C2_2_ domain of Siglec-7 was replaced with that of Siglec-9, despite the proteins sharing a sequence identity of 83%. The transduction of the cell death signal could be narrowed down to the presence of 4 amino acids of Siglec-7: W288, T289 and S292, located in the CE-loop, and the nearby L304. None of these residues are conserved in Siglec-9-C2_2_ with L287, S288, and G291 (Fig. 2A), whereas W288 in Siglec-7 is conserved in Siglec-10-C2_2_ (W288 in Fig. 2B) and Siglec-10-C2_3_, as well as T289 in Siglec-10-C2_3_. W288 in the CE-loop of Siglec-10-C2_2_ (Fig. 2B) corresponds to W2 in the both Sig10-C2_2_ and Sig10-C2_3_ peptides, where the residue was shown to play a role in binding to AOC3 by both STD-NMR and in silico experiments. Based on these results, it is tempting to speculate that the C2 domains of Siglec-10 might also have a physiological role on the leukocytes expressing Siglec-10 as a surface receptor.

## Conclusion

To conclude, we show through an integrative structural approach that AOC3 binds to the short 10-residue circular peptides derived from the CE-loops of the C2 domains in Siglec-9 and Siglec-10. The different methods used are in agreement with each other and strengthen the validity of the findings compared to purely in silico or in vitro experiments. Our results highlight the importance of the specific residues in the peptides and provide hints for their physiological relevance in the Siglec-9 and Siglec-10 proteins. Based on our study, the WRG motif in Sig9-C2_2_ is critical for its interaction with AOC3. The MST studies of Sig9-C2_2_ and STD-NMR of the very similar Sig9-phage confirm that they bind in vitro. The shortened Sig9-C2_2_ peptide still exhibits the characteristics, which we identified to be the most critical for good binding to AOC3. Its WRG motif has a tryptophan residue in position 4 and an arginine residue in position 5, which are respectively predicted to interact with the specificity pocket and the catalytic aspartate D386 in AOC3. The fact that the WRG motif of Sig9-phage and Sig9-C2_2_ is not conserved in any other C2_2_ or C2_3_ domains of Siglecs [4] further supports its importance.

**Fig. 7.**
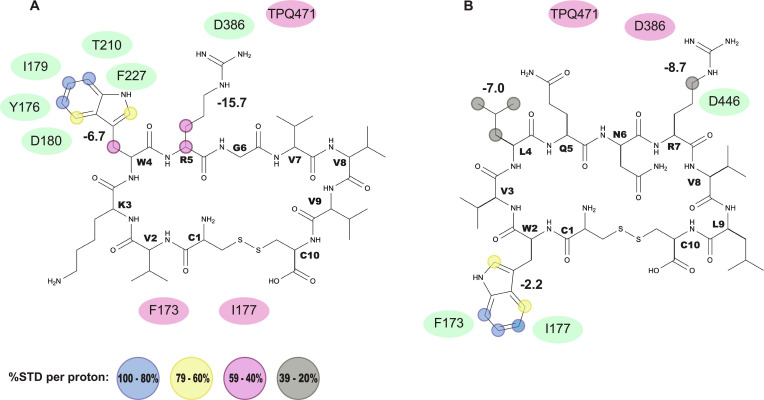
Consensus binding modes for (A) Sig9-phage and (B) Sig10-C2_2_. The 2D representations of the peptides are laid out, with the STD intensities shown according to the legend and interacting residues from the distance measurements in light green ovals. Pink ovals are non-interacting residues included to orient the peptides relative to the active site. The MMGBSA values of select residues are typed out in bold text next to the respective residues.

A consensus binding mode for Sig9-phage and Sig10-C2_2_ that integrates the docking with MMGBSA results and STD-NMR is laid out in Fig. 7. The Sig10-C2_2_ peptide, which corresponds to the Sig10-phage peptide previously known to bind AOC3 [28], displays similar *K*_D_ as Sig9-phage and Sig9-C2_2_ and shows a similar involvement of the tryptophan residue in binding to AOC3, but molecular docking predicts that instead of binding to the specificity pocket as W4 in the Sig9 peptides, W2 in Sig10-C2_2_ points outward interacting with I177 and F173.

In conclusion, the short 10-residue Sig9-C2_2_ peptide could be a better alternative for the longer Sig9-C2_2_ peptide currently used in PET imaging of inflammation [22,29,30]. Although the Siglec-10-C2-derived peptides do bind to AOC3, they are not ideal for achieving the tightest possible binding to AOC3 compared to the Siglec-9-C2_2_-derived peptides. Thus, our results with the Siglec-9- and Siglec-10-derived peptides imply differences in the biological functions of Siglec-9 and Siglec-10 and their interactions with AOC3.

## Data Availability

The data that support the findings of this study are available from the corresponding author upon reasonable request.
