# Supplementary material for: Exploring the Binding Specificity of Siglec-Derived Peptides to Human Primary Amine Oxidase Using an Integrative Approach to Ligand Epitope Mapping
Source: Comput Struct Biotechnol J. 2026 Aug 5;35(1):0195. doi: 10.34133/csbj.0195 (PMC13438033; doi:10.34133/csbj.0195)
Supplement: Supplementary file 1 [file csbj.0195.f1.docx]

Supplementary Materials

**Supplementary materials and methods**

**STD-NMR negative control experiment.**

To verify that the STD-effect observed between Siglec peptides and AOC3 is due to a specific interaction and not just the peptides binding to any protein, a control experiment was made with 500 µM of Sig10-C23 in the presence of 5 µM bovine serum albumin (BSA). The STD spectrum was acquired in the same way as the other STD spectra. The spectra show (Figure S3) that there is some STD-effect observable for the valine γ-protons and leucine δ-protons with chemical shifts around 0.8 ppm, but no STD-effect is observable at other regions. Notably, the aromatic protons of the tryptophan residue, with chemical shifts around 7 ppm, which show the closest interaction with AOC3, do not show any interaction with BSA.

**Supplementary figures**

***Fig. S1.*** *The fragment of pyridazinone inhibitor (PDB ID: 4BTY) used in docking experiments. The modified ligand lacks the two rings (grey), which point outward of the AOC3 active site. Protein structure is shown in light blue (chain B) and beige (chain A) and the ligand used in docking in pink.*

***Fig. S2.*** *Distances (Å) between residues of docking representatives and nearby AOC3 residues during MDs. R interactions are shown in shades of blue, W interactions are shown in shades of orange and Q4 (Sig10-C2_3_ peptide) is shown in green. (a) Sig9-PET peptide, (b) Sig9-phage peptide, (c) Sig9-C2_2_ peptide, (d) Sig10-C2_3_ peptide, (e) Sig10-phage peptide, (f) Sig10-C2_2_ peptide.*

***Fig. S3.*** *Negative control STD-NMR spectra of Sig10-C2_3_ in the presence of BSA. The black spectrum is the off-resonance spectrum, and the red spectrum is the STD-spectrum, magnified for clarity.*

**Supplementary tables**

| **Peptide** | **Measurement 1** | **Measurement 2** | **Measurement 3** | **Measurement 4** | **Average** |
| --- | --- | --- | --- | --- | --- |
| Sig9-phage | 17.0 | 19.7 | 17.4 | 32.30 | 21.6 |
| Sig9-C2_2_ | 17.7 | 17.3 | 18.5 | - | 17.9 |
| Sig10-C2_2_ | 10.6 | 14.0 | 21.2 |  | 15.3 |

***Table S1.*** *Results of individual K_D_ measurements between AOC3 and siglec peptides, all values given in µM.*

| **Sig9-phage** | | | **Sig10-C2_2_** | | | **Sig10-C2_3_** | | |
| --- | --- | --- | --- | --- | --- | --- | --- | --- |
| Residue | Proton | Shift (ppm) | Residue | Proton | Shift (ppm) | Residue | Proton | Shift (ppm) |
| V2 | α | 4.40 | W2 | α | 4.96 | W2 | α | 5.06 |
| V2 | β | 2.04 | W2 | β2 | 3.22 | W2 | β2 | 3.31 |
| V2 | γ | 0.91 | W2 | β3 | 3.33 | W2 | β3 | 3.37 |
| K3 | α | 4.80 | W2 | δ1 | 7.18 | W2 | ε3 | 7.56 |
| K3 | β2 | 1.72 | W2 | ε3 | 7.58 | W2 | η2 | 7.15 |
| K3 | β3 | 1.66 | W2 | η2 | 7.20 | W2 | ζ2 | 7.41 |
| K3 | δ | 1.57 | W2 | ζ2 | 7.46 | W2 | ζ3 | 7.11 |
| K3 | ε | 2.87 | W2 | ζ3 | 7.10 | T3 | α | 4.91 |
| K3 | γ | 1.25 | V3 | α | 4.44 | T3 | β | 4.08 |
| W4 | α | 4.89 | V3 | β | 1.96 | T3 | γ2 | 1.10 |
| W4 | β2 | 3.25 | V3 | γ1 | 0.89 | R5 | α | 4.04 |
| W4 | β3 | 3.23 | V3 | γ2 | 0.86 | R5 | β2 | 1.86 |
| W4 | δ1 | 7.29 | L4 | α | 4.48 | R5 | β3 | 2.00 |
| W4 | ε3 | 7.68 | L4 | β | 1.62 | R5 | δ | 3.24 |
| W4 | η2 | 7.26 | L4 | δ1 | 0.99 | R5 | γ | 1.64 |
| W4 | ζ2 | 7.49 | L4 | δ | 0.93 | G6 | α2 | 3.64 |
| W4 | ζ3 | 7.18 | L4 | γ | 1.56 | G6 | α3 | 4.11 |
| R5 | α | 3.71 | Q5 | α | 3.93 | Q7 | α | 4.52 |
| R5 | β2 | 1.67 | Q5 | β2 | 2.13 | Q7 | β2 | 1.98 |
| R5 | β3 | 1.42 | Q5 | β3 | 2.18 | Q7 | β3 | 2.05 |
| R5 | δ2 | 2.80 | Q5 | γ2 | 2.34 | Q7 | γ2 | 2.29 |
| R5 | δ3 | 2.73 | Q5 | γ3 | 2.35 | Q7 | γ3 | 2.35 |
| R5 | γ2 | 1.00 | N6 | α | 4.40 | V8 | α | 4.26 |
| R5 | γ3 | 0.63 | N6 | β2 | 2.89 | V8 | β | 1.90 |
| G6 | α2 | 4.14 | N6 | β3 | 2.94 | V8 | γ1 | 0.85 |
| G6 | α3 | 3.61 | R7 | α | 4.45 | V8 | γ2 | 0.75 |
| V7 | α | 4.30 | R7 | β2 | 1.81 | L9 | α | 4.49 |
| V7 | β | 2.10 | R7 | β3 | 1.79 | L9 | β2 | 1.37 |
| V7 | γ | 0.98 | R7 | δ | 3.13 | L9 | β3 | 1.47 |
| V8 | α | 4.24 | R7 | γ2 | 1.63 | L9 | δ1 | 1.10 |
| V8 | β | 2.17 | R7 | γ3 | 1.61 | L9 | δ2 | 0.84 |
| V8 | γ | 0.93 | V8 | α | 4.34 | L9 | γ | 1.35 |
| V9 | α | 4.14 | V8 | β | 1.97 |  |  |  |
| V9 | β | 2.05 | V8 | γ1 | 0.90 |  |  |  |
| V9 | γ | 0.93 | V8 | γ2 | 0.87 |  |  |  |
|  |  |  | L9 | α | 4.46 |  |  |  |
|  |  |  | L9 | β | 1.56 |  |  |  |
|  |  |  | L9 | δ1 | 0.86 |  |  |  |
|  |  |  | L9 | δ2 | 0.82 |  |  |  |
|  |  |  | L9 | γ | 1.55 |  |  |  |

***Table S2.*** *Chemical shifts of amino side chain protons. Measurements done in 1x PBS, pH 7.4, prepared in D_2_O.*
